# Assessing the continuum between temperament and affective illness: psychiatric and mathematical perspectives

**DOI:** 10.1098/rstb.2017.0168

**Published:** 2018-02-26

**Authors:** William Sulis

**Affiliations:** Collective Intelligence Laboratory, Department of Psychiatry and Behavioral Science, McMaster University, 92 Bowman Street, Hamilton, Ontario, Canada

**Keywords:** temperament, mental illness, dynamics, classification, time series, methodology

## Abstract

Temperament of healthy people and mental illnesses, particularly affective disorders, have been conjectured to lie along a continuum of neurobehavioural regulation. Understanding the nature of this continuum may better inform the construction of taxonomies for both categories of behaviour. Both temperament and mental illness refer to patterns of behaviour that manifest over long time scales (weeks to years) and they appear to share many underlying neuroregulatory systems. This continuum is discussed from the perspectives of nonlinear dynamical systems theory, neurobiology and psychiatry as applied to understanding such multiscale time-series behaviour. Particular emphasis is given to issues of generativity, fungibility, metastability, non-stationarity and contextuality. Implications of these dynamical properties for the development of taxonomies will be discussed. Problems with the over-reliance of psychologists on statistical and mathematical methods in deriving their taxonomies (particularly those based on factor analysis) will be discussed from a dynamical perspective. An alternative approach to temperament based upon functionality, and its discriminative capabilities in mental illness, is presented.

This article is part of the theme issue ‘Diverse perspectives on diversity: multi-disciplinary approaches to taxonomies of individual differences’.

## 1. Introduction: the concept of continuum

The concept of temperament is frequently conflated by psychologists with the concept of personality. Temperament refers to neuro-chemical and biological properties of nervous systems. Personality, on the other hand, is a socio-cultural construction acquired through experience and learning. Animals and infants have temperament, but personality emerges through life experience. Traditionally, starting from Kant, temperament traits have been broadly classified into emotionality (related to emotional regulation) and ‘activity’ (related to the energetic and orientational aspects of the regulation of activity).

It has been conjectured that temperament and mental illness, particularly affective disorders, lie along a continuum of neurobehavioural regulation [1–5]. If confirmed, this would provide an opportunity to gain an understanding of both classes of behaviour through their coupling to these regulatory systems. It would benefit researchers to have taxonomies for classifying temperament and mental illness which couple to salient dynamical and functional aspects of this continuum. These taxonomies should be both sensitive and responsive to changes in the dynamics of the neurobehavioural regulatory systems, emerging as changes in temperament and/or the appearance or remission of states of mental illness. Moreover, if the classifications of temperament and mental illness reflect dynamical interrelationships between these regulatory systems, then dispositions or alterations in these interrelationships should be reflected in correlations or systematic changes between temperament patterns and the presence or absence of mental illness.

The most popular models of temperament currently in use base their taxonomies on factor analytic analyses of correlations among common language descriptors of behaviour. These descriptors arose within the native culture, presumably in the service of ecological needs, but lack definitional rigour and any deep connection to modern concepts of dynamics [6]. There is no reason, *a priori*, to believe that such descriptors or the derived factors should bear any relationship to the dynamics of the regulatory systems (for example, as happened with the Five Factor model [7]). Taxonomies of mental illness have also faced criticism in recent years for being too strongly based on poorly discriminating symptom clusters organized primarily by expert consensus [1,8]. The development of taxonomies arising from the continuum concept offers the possibility of a better alignment between the taxonomic categories and the putative underlying dynamics. This might better inform our understanding of predisposition, as well as individual differences in illness presentation, course, therapeutics and treatment response. Several lines of evidence support this continuum concept. First, there are many symptoms of mental illness that possess an enduring nature and which bear a striking resemblance to temperament traits. For example, the *Diagnostic and Statistical Manual of Mental Disorders*, Fifth Edition (DSM-5) criteria for major depression of fatigue, concentration and worry are similar to the traits of endurance, plasticity and self-confidence, as well as neuroticism. Second, associations have been found between certain forms of mental illness and particular temperament traits [9]. More will be described later. Nery *et al*. [10] have shown that some of these associations may be state-dependent effects, appearing in the presence of an illness state and reverting to baseline following remission of the illness. Third, neurobehavioural research has demonstrated significant overlap between the regulatory systems thought to be involved in affective illness and those involved in temperament. These include monoamine, acetylcholine and neuropeptide systems [11].

The continuum should not be thought of a simple linear continuum with temperament and mental illness at opposite poles. Neurobehavioural regulatory systems are interdependent complex adaptive systems capable of expressing a broad range of (emergent) dynamics. It is this space of possible dynamics that forms the continuum, and temperament and mental illness occupy different regions of this space. Two metaphors might help to illuminate this idea.

My background^1^ allows me to address these issues from both a mathematical and a psychiatric perspective, to which I now turn.

## 2. Mathematical perspective

To a mathematician, like me, it is rather surprising, if not shocking, to observe that psychologists have a tendency to rely upon statistical methodologies to create, confirm or disconfirm their taxonomic theories. Other sciences, mathematics included, instead search for underlying principles of their taxonomies which universally can be applied to a broad range of their taxonomic objects, irrespective of any particular instantiation. Statistics cannot substitute for critical observation and thinking. Mathematicians themselves spend a great deal of time categorizing, classifying and creating taxonomies of these universals in taxonomies of mathematical functions, numbers, etc., without the use of statistics. The presence of such universal features within the continuum could provide the basis for the development of a taxonomy of the continuum.

This, in turn, could inform the creation of taxonomies for temperament and mental illness. Two metaphors may aid in understanding this suggestion. The formal theory of computation provides the first metaphor [12]. One universal concept is the distinction between hardware and software. Hardware refers to the physically realized dynamical system that performs the computation. Software is symbolic and informative. It possesses a dual nature—as data for computation and as instructions directing the dynamics of the hardware to compute. To be efficacious, software must be realized as states of the hardware which serve as (internal) control parameters guiding the dynamics of the hardware. For simplicity and conceptual clarity, ignore the environment and represent the hardware as an iterated function system, each configuration having the functional form, *T_P_*(*X, D*), where *X* is a vector representing autonomous states of the hardware, *D* is a vector representing data states and *P* is a vector of states representing instructions, considered as control parameters. These control parameters alter the dynamics expressed by *T_P_* according to the needs of the computation. The action of *T_P_* (*X, D*) is to move to a new hardware configuration, *T_P′_* (*X′, D′*). For fixed *P*, we can write *T_P_* (*X, D*) = (*X′, D′*), so properly *T_P_* (*X, D*) refers to a family of functions.

Hardware constrains the types of software that can be implemented and the availability of resources. In computers, software is determined by the environment and the hardware is protected, but in nervous systems, software (psychological states) arises in an emergent manner from interactions with the internal and external environments. Causal influences appear to act both upward and downward [13]. Software may alter hardware, through, for example, long-term potentiation or gene expression. The transition may then take the form *T_P_ (X, D*) → *M_P′_* (*X′, D′*), resulting in a new hardware function *M*. In the course of experience, the system will undergo a succession of transitions 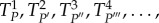 resulting in a superfamily (i.e. coherent collection of families) of dynamical systems.

It seems natural to associate temperament, defined as the biological basis of individual differences, with the function family *T_P_* since hardware corresponds to biology. *T_P_* disposes the hardware to behave in response to (*X, D*) just as temperament disposes a person to behave in different contexts. Since temperament refers to stable characteristics, the same temperament should be associated some superfamily 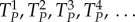 of dynamical systems. The mathematician would then search for characteristics, signatures and invariants of these superfamilies, whether in formal descriptors of the functions and/or their dynamics, or symmetries connecting the functions forming a superfamily or the geometrical objects (such as fixed points, attractors, repellors, strange attractors, Cantor dust, stochastic webs) associated with the dynamics. One would also search for critical points, markers of stability or instability and so on. In particular, one would search for signatures in time series of behaviour that could be linked to these structural characteristics.

Temperament in humans is thought to be related to monoamine, acetylcholine and neuropeptide systems, which project to cortex, thus regulating behaviour. The cortex appears to have few projections back to these systems, leaving them relatively immune to experiential modification over time [14]. Thus, the superfamily 
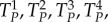
 which represents the entire nervous system, is expected to share some common features (comprising a smaller family of dynamical systems *R_P_*) which represent the contributions of these neurobehavioural regulatory systems. It is then natural to ask whether signatures of these regulatory systems can be detected in the observed time series.

To make this idea concrete, consider the familiar logistic map *f*(*x*) = *λx*(1 − *x*). Here, *x* is a datum, *λ* is a control parameter. Each such map corresponds to a single iterative function system. Allowing *λ* to smoothly change results in a family of iterated maps. Different intervals for *λ* correspond to iterated maps having similar dynamics, for example period 1, period 2, period 4, period 6, to odd period, intermittent, aperiodic and chaotic dynamics. These transitions form a bifurcation sequence in which the transitions occur as one stable region becomes unstable and transitions to a new stable regime with different dynamics. There is experimental evidence for the existence and prominent role of such dynamics in nervous systems [15–17]. They may be essential to psychological functions [16].

Mental illness could affect the dynamics by either altering the function *T_P_* or by expanding or restricting the spaces underlying *X, D* and *P*. The former changes would be viewed as biological, the latter as psychological, even though both must ultimately be expressed through states of the nervous system. As temperament is thought to be a long-term characteristic, this implies that it should be stable from a mathematical perspective. The transition from normality to mental illness would be expected to occur when the normal dynamic becomes unstable and a transition can occur. Thus, it is also important to look for signatures of stability and instability.

The continuum can therefore be understood as the global space of dynamical systems. Temperament and mental illness will correspond to certain superfamilies defined on this continuum. These superfamilies will possess mathematical characteristics and signatures that distinguish (and sometime interrelate) them. Any effective taxonomies of temperament and mental illness should find some correspondence with the taxonomy of these superfamilies. Knowledge of the mathematical taxonomy could thus help to inform and shape the taxonomies used for temperament and mental illness.

As an aside, efforts to extend the computational metaphor further to assert that brain function *is* computation appear rather misguided. It is not clear that the Turing limit suggested by computation theory applies to the brain and some models of neural networks explicitly state otherwise [18].

It is suggested that signatures of these dynamical structures should be sought in time series of suitably selected real behaviour. The choice of context must be informed by non-mathematical considerations. The most promising appears to be based on evolutionarily or ecologically salient functionality (discussed later).

The second metaphor provides some insight into the proper features to focus on in these time series. It is derived from meteorology. Three distinct time scales form the basis for studies in meteorology [19]. Short time scales from minutes to hours are characteristic of local weather patterns. Mid-range time scales of hours to weeks are characteristic of weather systems and seasonal patterns. Long-range time scales, extending over years and longer, are characteristic of climate. Individual behaviours are analogous to local weather, while mood states are analogous to weather systems. Temperament is analogous to climate. It is long term, constrains and disposes behaviour without determining the specifics of individual behaviours, just as climate does for local weather. Mental illness can manifest at all three time scales. Illness is akin to local storms, system-based storms or climate change [20].

Unfortunately, current taxonomies of adult temperament have mostly been based on common language descriptors of behaviour rather than direct observation. Some studies of childhood temperament, particularly those of Kagan [21], were based on direct observation of behavioural patterns in structured environments. Other childhood temperament researchers [22] used parental reports, but carried out direct observations to confirm the consistency and validity of those reports. The kind of data suggested above is thus scarce in the psychological literature, but some case reports have appeared in the psychiatric literature, particularly in regard to mood, as will be discussed later.

## 3. Challenges for multiscale complex systems analysis

It should be obvious that an analysis of the structure and dynamics of the continuum will require the use of multiscale analysis, both temporal and spatial [23]. If our taxonomies must classify superfamilies of dynamical systems within the continuum of neurobehavioural regulatory systems, then it is necessary to understand some of the known dynamical characteristics of these systems (and neural systems generally). There are several characteristics which are vitally important for understanding the dynamics of nervous systems as a whole and many of them pose serious challenges to multiscale mathematical modelling and statistical analysis. Most of these are ignored by researchers. Some of these will be discussed in the next section. Here, the focus is on the consequences of emergence within the nervous system.

Psychological phenomena are thought to be emergent from the chaotic nonlinear dynamics of the nervous system [24]. Phenomena emerge from the complex interplay between various levels within the nervous system and both the internal (somatic) and external environments. The notion of causation is perhaps better replaced with the idea of influence, because seldom does the behaviour of a single subsystem ever wholly determine that of another. There are also long-term processes of development and senescence at play. Formally, this implies that the phase space upon which the dynamical system acts is itself dynamic. This is profoundly challenging to model. Moreover, these emergent processes exhibit several key features: generativity, fungibility, metastability and contextuality.

Decades ago, Anochin [25] and Bernstein [26] noted that motor actions are constructed anew each time that they are performed. There is no simple relationship between neurons or neural systems and behavioural acts. The retrieval of memories [27] is also an act of construction, with each memory being formed from different associations among different neurons each time it is retrieved. Emotions too appear to be generated rather than being intrinsic features of the nervous system [28]. The construction of a memory or of a specific motor act is very much like the performance of a play, with different neurons and different neural pathways entering into each and every performance. Just as actors are fungible, so are the neurons that enter into these acts.

Closely related to fungibility is the phenomenon of metastability. A classic example involves the spatial maps supported by hippocampal place cells in rats [29]. Originally, it was thought that hippocampal place cells formed a one–one correspondence with locations within the rat's physical environment. More sophisticated real time recording techniques later showed that these correspondences changed as a result of the rat being moved to a new environment. Nevertheless, the rat, when returned to the original environment, could carry out previously learned spatial tasks with similar levels of success.

Context also plays a prominent role, manifesting in the phenomenon of *functional rewiring*, most notable in the lobster stomatogastric ganglion [30]. The connectivity among these 50 cells effectively changes as a result of circulating neurohumoral peptides and hormones secreted by the gut. This enables this small collection of cells to control all of the different functions needed for digestion, from ingestion to excretion. Functional rewiring is ubiquitous throughout the nervous system even in humans. This occurs because many synapses are based upon g-protein-coupled receptors. Unlike ligand receptors, which directly cause depolarization of the neural membrane (and possibly action potentials), g-protein-coupled receptors alter the responsiveness of the cellular membrane, effectively altering the dynamics of the neuron and the coupling between the participating neurons [31].

Contextuality does not only occur in relation to the environment. A century ago, Pavlov showed that temperament traits in dogs were coupled and emerged in a manner reminiscent of bifurcations in nonlinear dynamical systems [32]. Pavlov noted that some temperament traits emerged only when other temperament traits exceeded some threshold. It is difficult to imagine how correlational techniques such as factor analysis would discriminate between such emergent factors. The dimensions of factor analysis are supposed to exist *a priori*, not to emerge under selected conditions.

Generativity, fungibility, metastability and contextuality pose serious challenges for formal modelling of these phenomena. One approach uses dispositional cellular automata, whose dynamics involve alterations in the automaton rule associated with each individual cell in response to external stimulation. This roughly models the dynamical effect of g-protein-coupled receptors. Dispositional cellular automata exhibit the phenomenon of transient-induced global response synchronization (TIGoRS) [33]. This involves the appearance of a stable correspondence between random samplings of certain spatio-temporal transients input into the automaton and the subsequent responses of the automaton. This synchronization occurs at the global level between the automaton and the transients, and not between the cells of the automaton. When TIGoRS is present, there is a nonlinear relationship between the input sampling rate and the Hamming distance between the transient stimulus and the transient response of the automaton. This synchronization occurs in spite of the fact that the underlying rule space of the automaton is constantly changing. The presence of TIGoRS demonstrates that finite duration spatio-temporal transients are the proper dynamical states to be studied. In the presence of TIGoRS, the dynamical action on these transients causes the dispositional cellular automaton to act as a primitive stimulus-response system [33].

In addition, the nervous system is riddled with intrinsic randomness. The release of neurotransmitter by a neuron is random [34]. The response of a neuron to repeated presentations of the same stimulus appears to be stochastic [35]. The response of a population of neurons to a stimulus also appears to be stochastic [35]. There is evidence that this apparent randomness is not due to noise, but instead is an expression of deeper, possibly chaotic nonlinear dynamics [16,24]. Treating this variability merely as noise may eliminate the possibility of observing, analysing and understanding the true dynamics of the nervous system.

Multiscale analysis of complex adaptive systems is challenging, complex and subtle. Feedback may go in any direction: up, down, horizontally. Biopsychosocial systems may exhibit what Cohen & Stewart [36] termed ‘complicity’ and Laughlin [37] termed ‘stable protection’. In this event, emergent phenomena hide or mask underlying microscale dynamics. The converse is also possible. Cohen and Stewart called this ‘simplexity’, while Laughlin called it the ‘deceitful turkey effect’. This is a situation in which emergent phenomena create an impression of a stable microscale dynamics where none actually exists. Observation and analysis of a system at only a single scale will never detect these possibilities. It is easy to be misled.

Situations may exist in which the dynamic at one level is effectively decoupled from that at neighbouring levels and so it can be observed and analysed as if it existed in isolation. Some psychological states may indeed be like this. There may be other situations in which what occurs at one level depends intimately on what is happening at adjacent levels, up or down. In such a case, it will be essential to have observations at these additional levels in order to understand what is transpiring at the level of interest. This is likely true of many illness states. Only a careful and thoughtful multiscale analysis offers any hope of detecting these situations and unravelling the nuances of the dynamics of nervous systems, and in particular the continuum and its relationship to emergent phenomena such as temperament and mental illness.

Can researchers truly believe that subjects can capture the nuances of this complex dynamics in their responses to a series of questions that ask whether some behaviour occurs ‘all of the time’, ‘most of the time’, ‘some of the time’ or ‘not at all’? Gottschalk *et al.* [38] were among the first to study time series of mood ratings. They showed that subjects' intuitive or common conceptions, such as normal mood is steady, bipolar mood cyclic, are really quite inaccurate.

## 4. Contextual probability

Contextuality, referred to in the previous section, has a profound effect on the dynamics of complex adaptive systems, such as nervous systems, one that goes to the very core of the structure of probability itself. Virtually all of modern probability and statistics is founded on the formal probability theory of Kolmogorov. In recent years, much as occurred in geometry, there have arisen competing models of probability theory, the most developed being contextual probability theory [39,40]. Khrennikov [41] has pointed out that there are conditions which must be met to ensure that a single joint probability distribution may be constructed when combining results across differing contexts (conditions). This is a deep consequence of contextuality. Forming such a distribution is a common construction when doing meta-analyses or when experiments are carried out involving multiple conditions as in the biomedical sciences. It is not well known that it is not always possible to do so.

Dzhafarov has presented the heart of this issue in a particularly cogent manner. Dzhafarov has described ‘Contextuality by Default’ [42]. Each random variable is associated with the quantity *q* being measured and the context *a* within which the measurement is made. He denotes each such variable 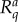. Consider two measurements, *q,q′* and two contexts *a,b*. For a fixed context *a*, the pair 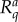 and 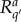 is termed a *bunch* and represents the collection of measurements associated with a specific context. It is reasonable to believe that such a pair is jointly distributed. For a fixed measurement *q*, the pair 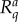 and 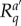 is termed a *connection* for *q*. A connection need not possess a joint distribution.

The most basic form of contextuality occurs when no joint distribution can be found for a connection. In such a case, they are said to be *inconsistently connected*. This is the situation of *contextuality by default*. It is quite common in the biopsychosocial sciences [43].

The fundamental difference between Kolmogorov and contextual probability lies in the way in which total probabilities are calculated. In Kolmogorov theory, one calculates the probability of an event *A* by summing over the conditions *B_i_* that give rise to *A.* Thus, *P*(*a*) = *Σ_i_ P*(*B_i_*)*P*(*A|B_i_*)*.* In developing contextual probability theory, Khrennikov began by modifying Kolmogorov's rule for total probability. Khrennikov explicitly accounts for context C, so that the total probability rule takes the form *P*(*A*)*_C_* = *Σ_i_ P*(*B_i_*)*_C_ P(A|B_i_*) *+* *2λ*(*A|B_i_,C*) (*Π_i_ P*(*A*)*_C_ P*(*A|B_i_*)^1/2^ where *λ* may be a trigonometric or a hyperbolic function (in Kolmogorov probability theory, *λ* = 0). The probability structure of quantum mechanics obeys this rule. The standard statistical tools used in psychology do not.

The techniques used to define and model dynamical systems in the setting of classical physics do not permit contextual probability. Quantum mechanics is a contextual probability theory, but it has limited use in psychology and psychiatry. New statistical tools need to be developed which can take contextuality into account. It is quite possible that much of our current knowledge which is derived from multiply conditioned data is inaccurate because of the failure to take contextuality into account when carrying out statistical analyses.

Statistical tools to address contextuality will only be effective if the data upon which they are applied has taken contextuality into account. Clinical experience has shown that the expression of temperament traits and of illness symptomatology is context dependent. Jung observed this in the expression of temperament more than a century ago. Insufficient attention has been paid to this in developing assessment tools, in interpreting observational data, in developing questionnaires and diagnostic tests. More attention needs to be paid to determining the impact that different contexts have on the expression of phenomenology, and to determining which contexts provide the most robust conditions for eliciting particular traits or symptoms. Kagan [21] implicitly considered this when basing his studies on observations under controlled conditions rather than relying on questionnaires.

The mathematical methods currently in use for describing biologically grounded dynamical systems do not easily capture features such as generativity, transience, fungibility, metastability and contextuality. Moreover, they do not describe dynamics obeying the rules of contextual probability. State spaces are given. The object of study is usually the flow of individual states. Components are assumed to be fixed. Processes of development in which the state space changes and the components that comprise the system change are from difficult to impossible to model in any but a cartoon fashion. There are novel techniques such as set valued calculus [44,45] that can describe some features of development, but they are far from being mainstream. An exciting development has been the creation of process-based models. These models started appearing in computer science many years ago and in quantum mechanics more recently. Two models that explicitly have generativity, transience and contextuality as fundamental features have appeared in quantum physics [39] and psychology [46]. The basic entities of both models are dynamical transients from which the entities of interest (fundamental particles, neural states, psychological states) are emergent. State spaces are generated (like the basic entities) and dynamic, so that they change over time. These models admit contextual probability structures (as well as standard Kolmogorov probability where the dynamics supports it). The task for mathematicians is to characterize and classify the dynamics of these models, to identify their invariants, symmetries and signatures, and to see to what degree these may be reflected in observed behaviour, whether in time series, as advocated here, or in correlations obtained from carefully selected experimental contexts.

## 5. Psychiatric perspectives

The literature on adult temperament reveals little use of direct observation or time-series methodologies. When psychologists do use time-series methodologies, they tend to use linear techniques [47], although a small number of researchers use nonlinear methods [48], and virtually none use detailed multiscale analysis. Mathematicians and physicists, however, have identified a wide range of salient characteristics and many different techniques for their detection and measurement [49]. There are phase space plots, recurrence plots, symbolic dynamics representations, correlation dimension, fractal dimension, Lyapunov exponents, Hurst exponents, BDS statistics, Renyi entropies, fluctuation distributions and spectra. Other measures include the number of turns, dwell times, variation, extremal values, crossing numbers. Each of these measures captures different dynamical characteristics of the time series. The goal is not to search for single measures but rather for a spectrum of measures which will serve to characterize different taxonomic categories, whether of temperament or of mental illness.

Psychiatrists have applied several time-series methods to the study of the temporal variation of mood, particularly in major depression and bipolar disorder, although all focus on a single scale level. The first such study appeared in Europe in 1975 [50]. Cluster techniques were applied to mood time series in the 1980s [51,52]. The first North American study was by Gottschalk, Bauer and Whybrow [38] who applied techniques from nonlinear dynamical systems theory. Since then, a wide variety of techniques have been used including Approximate entropy [53,54], Lyapunov exponents and correlation dimension [55], power laws [56], surrogate data and nonlinear forecasting [57], autocorrelation and cross-correlation [58] and others [55,57–62]. To date, the available evidence appears to support the presence of nonlinear or chaotic dynamics, but the source of this dynamics, whether due to intrinsic or extrinsic factors, has yet to be determined.

Approximate entropy was found in one study to increase in the 60 days prior to the onset of an episode of mania or depression, with the greater increase preceding mania [54]. Power law scaling was found in fluctuation distributions in time series from patients with depression and controls [56]. The exponent was higher (2.5) in controls compared with depressed subjects (1.7–1.9), suggesting an alteration in dynamics. Analysis of variation in time series was able to distinguish between stable and unstable groups of patients with bipolar disorder [60]. Another study used variability to distinguish bipolar patients from those with borderline personality disorder [61]. Several studies explored correlations between dynamical features of mood time series and treatment conditions using antidepressants or psychotherapy [63–65]. For example, one study used time-series analysis to distinguish between treatment responders and treatment non-responders among depressed patients treated with antidepressants. Correlations between dynamical features of mood time series and temperament traits have also been examined [66–69]. In one study, the analysis of the Hurst exponent of a worry time series appeared to correlate with temperament factors [62]. In another, the presence of a 7-day cycle in Positive and Negative affect in healthy volunteers was found [66]. These results are preliminary and few, but they do show the potential utility of using time-series analysis to shed light on the aspects of temperament and mental illness.

Psychiatry has been in the vanguard in attempting to develop more formal approaches to the description of psychiatric phenomena. Notable is the work of Mandell & Selz [70], who applied the language, concepts and methods of nonlinear dynamical systems theory to this task. They went beyond theory and applied these methods to the analysis of experimental data, thus demonstrating the usefulness of this approach. Their work is quite sophisticated mathematically, which may, in part, explain why it has not been embraced by the psychiatric community generally. They do not address temperament per se, nor do they consider the role of different time scales and contextuality. There have been a few subsequent attempts in psychology to apply nonlinear dynamical systems ideas to the study of personality [71,72].

There is a large literature exploring the connections between current temperament taxonomies and mental illness, with much of this research focused on temperament models and traits related primarily to emotionality, such as Negative Affect [1], Harm Avoidance [10], Neuroticism [73] and Depressive Affective Temperament [74]. Positive/negative affect models provide the basis for many categories of illness in the DSM-5, yet these same models of temperament appear to be very insensitive in differentiating between various types of mental disorders, especially between depression and generalized anxiety. Indeed in Watson's quadripartite model [9], major depression (MD) and generalized anxiety disorder (GAD) lie within a single factor of ‘distress disorders’. Anxiety [2,3] and depression [75] are both associated with higher scores on Neuroticism/Negative Affect scales. Neuroticism, however, appeared to be high not just in anxiety disorders but in many types of mental illness and therefore did not differentiate between mental disorders. These findings show that the scales measuring Neuroticism/Negative Affect in many current temperament models do not differentiate well between depression, generalized anxiety and other mental illnesses even in terms of the components of emotionality.

More importantly, far fewer studies investigated the coupling between non-emotionality temperament traits and mental illness, in spite of the fact that the DSM-5 considers a broad range of non-emotional symptoms: fatigue, poor attention and memory, dysfunction in sleep, appetite, psychomotor retardation, agitation, lethargy or restlessness. Most of these studies used scales related to Extraversion, Sensation/risk seeking or Self-directedness, but none related to dynamical aspects of behaviour or physical functioning. Some studies have found associations of low Extraversion/Positive Affect with depression [4] and generalized anxiety [5]. Depression has been linked to higher Behavioural Inhibition [5], so has generalized anxiety [4,5]. A decrease in Self Directedness has been noted in depression [7] and in generalized anxiety [76]. Thus, the inclusion of these non-emotionality scales still did not enable these models to differentiate between depression and generalized anxiety. Most of the models used in these studies have shown little or no ability to discriminate between MD and GAD.

A different paradigm is needed, one that provides a better connection to underlying dynamics. As the dynamics that produces behaviour must ultimately provide ecologically salient functionality for the person, this led to a search for models of temperament that capture concepts of functionality while being rooted in psychophysics or neurophysiology of the regulatory systems. One promising model is the neurochemically inspired Functional Ensemble of Temperament (FET) model [11]. This is an extension of Rusalov's activity-specific model of temperament [77,78]. The FET model organizes temperament traits in a 3 × 4 matrix categorized by functional aspects of human behaviour [11]. The 12 components within the FET include: nine domains (traits) regulating formal functional aspects of behaviour (endurance, dynamic and orientational) each assessed in three domains (intellectual, physical and social), together with three systems related to emotionality (Neuroticism, Impulsivity and Self-confidence). Temperament is assessed using the Structure of Temperament Questionnaire [78]. The FET model posits ensemble relationships between monoamine, acetylcholine, neuropeptide and opioid receptor systems as providing the neurobiology underlying temperament. It suggests that the continuum should be localized to the dynamics of these regulatory systems, at least based upon our current understanding. The model predicts that the presence of mental illness should result in differential effects on levels of temperament traits, akin to a spectrogram.

As a psychiatrist, I was particularly taken by the correspondence between almost all of the symptoms described in the DSM/ICD classifications and FET components. This raises the possibility of structuring future classifications of mental illness in line with the functional aspects of human behaviour as proposed by the FET framework. Several studies have been carried out to examine the coupling between FET temperament traits and mental illnesses and demonstrated the value of using this functional approach. The FET framework appeared to differentiate between several diagnostic categories [79–83] better than other temperament frameworks that have been used in psychiatry. For example, the main differences between MD and GAD were in Motor Endurance and Motor Tempo (only the FET model has such components), which were lowered in MD and unchanged in GAD. A lesser difference was in Neuroticism, which was elevated in both disorders, but more so in GAD. The FET framework was able to differentiate between comorbid depression and anxiety, MD and GAD, which has proved difficult to do using any other models. Comorbid depression and anxiety differed from the other two diagnoses by a dramatic decrease in aspects of behaviour regulated by the neocortex—Mental endurance (sustained attention), Plasticity, Probabilistic thinking and Impulse control.

These selective differences could not have been detected without the separation of traits according to physical, social and intellectual domains, or without separation of traits related to endurance, speed of integration of actions or type of behavioural orientation. Conflation of these domains, as happens with most other models, eliminates any possibility of detecting these differences. This is clear evidence of the value that the activity-specific approach brings to the study of adult temperament and to attempts to explore the continuum.

## 6. Conclusion

It has been conjectured that temperament and mental illness lie along a continuum of neurobehavioural regulation. This continuum is not a simple linear grading of measurements with temperament and mental illness along opposite poles. Instead, the continuum is a complex representation of different dynamical regimes of these neurobehavioural regulatory systems. Temperament and mental illness are expressed in different psychological domains and across a wide range of temporal scales. An understanding of this continuum and of its relationship to temperament and mental illness may inform the study of both categories of phenomena and putative linkages between them. This may, in turn, lead to a better understanding of mental illness and perhaps assist in the planning of individualized treatment.

In order to better understand this continuum, it is necessary to coordinate the development of temperament taxonomies with taxonomies of mental illness. This would reflect the structure of the continuum between these two sets of individual differences. In order to develop these taxonomies, greater attention needs to be paid to the conceptual analysis of experimental data from multiple disciplines. Taxonomies based on factor analysis and other statistically derived groupings do not provide any obvious resonance with the continuum underlying temperament and mental illness. They are not very useful in advancing our theoretical understanding of temperament and mental illness [82].

Furthermore, it is essential that we use the correct statistical tools when analysing data obtained from such complex phenomena as temperament and mental illness and for testing out theories and taxonomies aimed at understanding such behaviour. Otherwise, we risk perpetuating the same errors that currently plague biomedical science, causing significant harm personally and financially [84].

Taxonomies based on activity-specific and functional approaches seem promising [85]. More attention needs to be paid to carrying out direct observations of behaviour in salient contexts and analysing these data in ways that connect with the underlying dynamics.

## Endnote

1 MD, FRCPC (geriatric psychiatry), PhD (mathematics), PhD (theoretical physics).

## Data accessibility

This article has no additional data.
